# Picturing AIDS: Using Images to Raise Community Awareness

**DOI:** 10.1371/journal.pmed.0010043

**Published:** 2004-12-28

**Authors:** Edwin Mapara, David Morley

## Abstract

In Botswana, explicit color photos of people with AIDS have been used to spread knowledge, with the aim of saving lives

Southern Africa poses special problems for AIDS educators and health care workers. Because there is a strong tradition of oral communication in the region, written educational materials often do not have as much impact as the spoken word. We have found that using colour images of HIV/AIDS in a workshop setting to provoke discussion can be a useful alternative to more conventional, written materials. In this article, we discuss our experience of using such images to raise community awareness about the AIDS epidemic in Botswana.

## Who We Are

Teaching-aids At Low Cost (TALC) is a nongovernmental organisation that supplies cheap teaching aids and books to raise standards of health care and standards of living—especially in poverty-stricken areas—worldwide (http://www.talcuk.org/). The organisation has traditionally focused on developing countries, particularly sub-Saharan Africa and Asia. In recent years, TALC has become more global; it now distributes materials to more than 200 countries and sends educational materials on CD-ROM at no cost to health workers in developing nations.

In 1964, TALC was founded at the London School of Hygiene and Tropical Medicine as a way of providing low-cost colour transparencies to help students from resource-poor countries to teach after they returned home. By the early 1980s, nearly half a million transparencies were being sold at cost each year. Those who used them came to appreciate how important colour images could be, particularly amongst people who have grown up in societies where knowledge is spread primarily through oral communication and less use has been made of the written word.

Early on in the HIV/AIDS epidemic, we decided that our experience of distributing visual teaching materials could be used to spread information about this new pandemic, which was hitting African societies particularly hard. We produced four sets of 24 colour transparencies on HIV/AIDS, with a detailed accompanying text.

## Edwin Mapara: The Botswana Experience

Today, there are an estimated 260,000 people in Botswana living with HIV. This—in a country with a total population of 1.6 million—gives Botswana a prevalence rate of 36.5%, the second highest in the world after Swaziland [1].

As a medical student in Zambia in 1985, I studied patients with Kaposi's sarcoma. The consultant in charge appreciated that this was due to HIV infection, but when she started to acknowledge this publicly, she was strongly censored by the existing authorities and was almost forced to leave the country. I realised that if this kind of denial persisted, the epidemic would spread more widely and would become an even greater disaster. I wanted to try to bring home to both the authorities and the African people the truth about the spread of the disease and the need for fundamental changes in sexual behaviour.

In 1990, on qualifying, I took up a post at the Athlone Hospital, a 175-bed district hospital in the Lobatse region of Southern Botswana. I joined other health workers who shared my concerns. We started the Athlone Anti-AIDS Project to address HIV prevention and care both in the hospital and in the wider community. We began to have organised discussions with local people about HIV/AIDS. The response we heard was often, “You talk about this terrible disease, which may affect us, but show us a patient”. This is how we came to use a set of slides from TALC, in a teaching programme that the Ministry of Health in Botswana called “radical and insensitive”.

**Figure 1:**
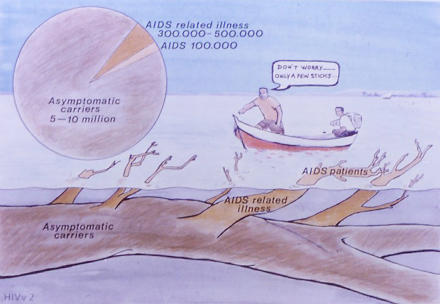
Don't Worry—Only a Few Sticks This slide is used in workshops to show that while we only see a handful of patients with symptomatic HIV, many more of us are HIV positive and are infecting others; we do not know our HIV status, since we have not been tested and we have no symptoms. (Illustration: TALC)

We emphasised the essential messages about AIDS prevention by using coloured pictures of black Africans. These pictures included explicit images of ulcers on a penis and a vagina. The slides included clinical manifestations of HIV/AIDS (such as herpes zoster and Kaposi's sarcoma) and other sexually transmitted diseases, images that explained the basic virology and transmission of HIV (Figure 1), and images about HIV prevention (such as condoms) and care (such as caring for orphans infected by HIV/ AIDS).

Showing these pictures to local people was hugely controversial. For example, some elderly participants walked out when they saw the explicit pictures. Some community members approached local political counsellors to voice their concerns about a “decay of culture” and a “lack of respect”. Some parents did not want their children to see the images at all, because “they would corrupt their morals and young minds” and would encourage children to “experiment with sex”. As the team leader, I was fined chickens on several occasions by local chiefs and elders for the “crime” of showing these explicit TALC slides.

The government gave our teaching project very little support in the early days; we were even cautioned by the highest authorities at the Ministry of Health. The Church, too, wanted nothing to do with our programme of “loose morals”.

Despite these obstacles, over the next ten years we held over a hundred workshops, which eventually involved all government departments and levels of society in Botswana. Today, we are still using the same pictures. In 2000, the United Nations Development Programme declared Athlone Hospital's initiative as one of the “best practices” in Botswana [2], and it is being replicated nationwide. TALC slides have been shown from the pulpits of churches, and community members will ask for the colour pictures specifically when the team is invited to lead a workshop.

Given the terrible impact that AIDS has had on the community, the same community members who once resisted our teaching project ask us angrily why doctors were not sufficiently aggressive in using pictures in the early days of AIDS. One telling statement made in a workshop was: “you doctors are to blame for what has happened to Africa, and particularly to our children. You should have done this ten years ago before one quarter of the population became infected. The blood of our children, who have died, rests on your heads”.

## Making the Best Use of Pictures

In Botswana, I used a slide projector and occasionally a mobile electrical generator, but such equipment is not widely available in most African countries. As an alternative to using colour slides, TALC has developed a folded A4 (210 mm × 279 mm) sheet with 12 colour images as a way of presenting the important messages about HIV/AIDS. This leaflet is available on request; E-mail: info@talcuk.org (or mail TALC, P. O. Box 49, St. Albans, AL1 5TX, United Kingdom).

In our experience, the slides or leaflet work best if you can get the participants to sit in small groups for discussion. Each group should have at least one set of pictures. In your introduction, mention that to talk about sex or death is not taboo in a world of AIDS. Encourage active participation by all.

Show one picture at a time—”let the picture talk”—and do not initially look at the accompanying text. Ask the participants to describe what they see in English or in the vernacular. Encourage participants to work out for themselves what the message is in the picture. Discuss all the possible answers. Then look at the text that accompanies the picture. Provide an answer built from the participants' words. If appropriate, ask people about their own relevant experiences. Finally, ask the participants to pin the pictures to the wall, making sure that each picture is put up by a different participant. Revise the lessons learned at the end of the session. Revise again, weeks later, if possible. At the end of the day, the participants should be able to say, “we did it by ourselves”.

The pictures show examples of how HIV/AIDS can affect people. They must not be thought of, or used, as a way to diagnose HIV/AIDS in participants or their relatives or friends. Emphasise to all participants that if they have any reason to suspect that they (or anyone they know) have HIV/ AIDS, they should attend a clinic where trained health workers can help them.

## Conclusion

For people in Botswana, “seeing is believing”. Written descriptions are often not enough; showing pictures of herpes zoster, syphilis ulcers, or tuberculosis lymphadenopathy can be a powerful teaching tool. Once the initial shock is overcome, these colour pictures offer a straightforward way to demonstrate the realities of the disease far and wide.

## Abbreviation

TALC: Teaching-aids At Low Cost
